# Biological Consequences of Single and Combined Exposure to Magnetite–Chitosan Nanocomposite with Adsorbed Cobalt (II) in *Danio rerio*

**DOI:** 10.3390/biology15080624

**Published:** 2026-04-16

**Authors:** Sergej Šemčuk, Danguolė Montvydienė, Renata Butrimienė, Aida Bradauskaitė, Galina Lujanienė, Martynas Talaikis, Kęstutis Mažeika, Vidas Pakštas, Justas Lazutka, Živilė Jurgelėnė

**Affiliations:** 1 State Research Institute Center for Physical Sciences and Technology (FTMC), Savanorių Ave. 231, LT-02300 Vilnius, Lithuania; galina.lujaniene@ftmc.lt (G.L.); martynas.talaikis@ftmc.lt (M.T.); kestutis.mazeika@ftmc.lt (K.M.); vidas.pakstas@ftmc.lt (V.P.); 2 State Scientific Research Institute Nature Research Centre (NRC), Akademijos St. 2, LT-08412 Vilnius, Lithuania; danguole.montvydiene@gamtc.lt (D.M.); renata.butrimiene@gamtc.lt (R.B.); aida.bradauskaite@gamtc.lt (A.B.); 3 Institute of Biotechnology, Life Sciences Centre, Vilnius University, Saulėtekio al. 7, LT-10257 Vilnius, Lithuania; justas.lazutka@bti.vu.lt

**Keywords:** magnetite–chitosan, metals, contamination, freshwater fish, combined effects

## Abstract

Magnetic nanocomposite sorbents are increasingly explored for the remediation of metal-contaminated waters; however, high abiotic removal efficiency may not always translate into biological safety. The present study evaluated the single and combined effects of dissolved cobalt (II) ions and magnetite–chitosan nanocomposites (MCN) in zebrafish (*Danio rerio*) embryos and larvae. MCN (30 wt.% Fe_3_O_4_) were synthesized via co-precipitation and crosslinking and physiochemically characterized. Adsorption experiments conducted in fish incubation medium demonstrated the efficacy of divalent Co removal and were well described by the Langmuir isotherm model, with a maximum experimental capacity of 20.08 mg g^−1^. The biological endpoints encompassed survival, hatching, heart rate, locomotor behavior, and oxidative stress biomarkers in early-stage zebrafish. The presence of cobalt (II) was found to result in a reduced hatching success rate, the induction of persistent bradycardia, and the occurrence of oxidative stress, as evidenced by a decline in SOD activity and an increase in H_2_O_2_ and MDA levels. The study found that MCN alone did not lead to mortality or increase peroxide levels or lipid peroxidation, although a modest decrease in SOD activity was observed. In contrast, combined exposure to cobalt and MCN resulted in significant delayed mortality (>85% at 96 h) and early neuromotor impairment. These findings indicate that high abiotic sorption efficiency alone does not guarantee reduced biological toxicity when nanomaterial–metal interactions occur. Consequently, safety assessments of remediation nanomaterials should explicitly consider nanomaterial–metal interactions and developmental stage-specific biological responses.

## Simple Summary

Cleaning metal-polluted water is an important environmental goal. Researchers are developing magnetic materials made from iron oxide and chitosan, a natural polymer, to reduce harmful metals in water because these materials can easily be recovered with a magnet. However, improving metal removal under laboratory conditions does not automatically guarantee the safety of the material for living organisms. In this study, we examined the effects of dissolved cobalt (II) ions (a potentially toxic metal) and magnetite–chitosan nanocomposites on developing zebrafish, a small freshwater fish commonly used in safety testing. It was found that Co (II) reduced hatching success, slowed heart rate, and caused stress-related symptoms in the fish. The nanocomposite did not cause mortality but slightly altered an important protective enzyme. When cobalt and the nanocomposite were present together, fish survival decreased sharply and early movement challenges were observed, even though the nanocomposites were capable of binding and partially removing cobalt from the water. These findings demonstrate that improved metal removal efficiency does not necessarily ensure biological safety under exposure scenarios. Interactions between metals and nanomaterials, especially during early life stages, must be carefully evaluated to ensure that water treatment technologies are environmentally safe.

## 1. Introduction

Engineered nanocomposites are increasingly being developed for the remediation of metal-contaminated aquatic systems, with performance typically assessed through abiotic metrics such as sorption capacity, binding kinetics, and reusability under controlled chemical conditions [1,2,3,4]. Among these materials, chitosan-based and magnetically retrievable nanocomposites have attracted substantial attention due to the strong metal-chelating capacity of chitosan and the practical advantages of magnetic separation and recovery [5,6,7,8]. Magnetite–chitosan nanocomposites (MCN), typically consisting of an Fe_3_O_4_ or γ-Fe_2_O_3_ core coated with chitosan, exemplify this class of materials and are frequently proposed as environmentally friendly platforms for the removal of dissolved metals via adsorption and complexation mechanisms. However, a key unresolved question remains: does high sorption efficiency in abiotic systems translate into meaningful toxicological protection in living organisms? Or could nano–metal interactions, formed during sorption, persist in exposure media and modulate metal uptake, toxicity, or biodistribution [9,10,11]?

This discrepancy arises from the difference between simplified abiotic sorption assays, which offer a reduced complexity, and the complex, organism-mediated microenvironments in which biological exposures occur. Within such systems, nanoparticle–metal complexes have been observed to interact with epithelial interfaces, perturb ionoregulatory processes, modify metal speciation, and induce oxidative stress [12,13]. Furthermore, the polycationic character of the polymer could enhance mucosal adhesion, while the iron oxide core has the potential to catalyze Fenton-like reactions under physiological conditions, thereby further amplifying the redox imbalance [14]. Consequently, these processes challenge the prevailing assumption that chemical removal directly translates into biological safety.

Cobalt (Co) is a trace metal of emerging concern in aquatic systems, particularly near mining or industrial discharge sites, where concentrations can reach 1–100 µg L^−1^ [15]. Although essential at trace levels, elevated cobalt (II) concentrations can induce oxidative stress, developmental toxicity, cardiotoxicity, and reduced survival in aquatic organisms [16,17,18,19,20]. The early life stages of fish are particularly sensitive to redox imbalances due to the rapid organogenesis and immature antioxidant defense systems characteristic of this developmental stage. This increased susceptibility to redox imbalances and physiological disruption can have deleterious effects on fish.

MCNs have been the focus of extensive research due to their potential as Co (II) sorbents. This is largely attributable to the high affinity of chitosan for divalent metal ions, as well as the magnetic recoverability of the core structure [2,21]. Evidence from in vivo freshwater fish models indicates that metal-binding nanomaterials have the capacity to reduce metal bioavailability and mitigate associated physiological and histopathological damage. For instance, biogenic magnetite nanogels (Fe_3_O_4_) have been shown to significantly alleviate lead (Pb)-induced organ dysfunction, oxidative stress, tissue injury, and metal accumulation in African catfish, thereby improving survival and antioxidant status [22,23]. In a similar manner, magnetite-based silica nanocomposites and aqueous silica nanoparticles have been observed to decrease Pb^2+^ uptake and mitigate oxidative, genotoxic, immunological, and histopathological alterations under single and combined metal exposure scenarios [24,25].

However, emerging evidence from early life-stage studies in zebrafish (*Danio rerio*) suggests that chitosan-based and magnetic nanomaterials are not biologically inert, even in the absence of co-exposed metals. Zebrafish embryo assays conducted under OECD TG 236, a widely recognized testing platform that is regarded as both sensitive and integrative, have revealed pronounced toxicity in chitosan-based nanomaterials that is dependent on both coating and formulation [26,27,28]. Chitosan-coated superparamagnetic iron oxide nanoparticles (SPIONs) have been observed to induce complete mortality at elevated concentrations and to elicit substantial alterations in locomotion, thigmotaxis, and escape responses [29]. Non-magnetic chitosan nanoparticles have also been shown to induce sublethal yet biologically relevant effects, including reduced liver size and altered light–dark behavior, even in the absence of malformations or lethality [30]. The developmental toxicity of chitosan nanoparticles is size-dependent; smaller nanoparticles have been shown to generate higher levels of reactive oxygen species (ROS) and to induce more severe functional impairments in comparison to larger counterparts [31]. Furthermore, the biological activity of co-delivered compounds is modulated by chitosan-based nanocarriers, which have been observed to affect acetylcholinesterase activity and locomotor behavior [32]. However, certain formulations, such as chitosan/ZnO nanocomposites, demonstrate low acute toxicity even at elevated concentrations, thereby emphasizing the formulation-specific nature of biological outcomes [33].

A mechanistic precedent for nanoparticle-mediated modulation of cobalt toxicity is provided by studies showing that selenium nanoparticles can attenuate CoNP-induced oxidative stress, apoptosis, muscular injury, and locomotor deficits in zebrafish larvae [34]. Despite the utilization of particulate cobalt as opposed to ionic cobalt (II) and the exclusion of magnetic chitosan systems, the findings indicate that nanomaterials possess the capacity to modify metal toxicity through mechanisms that extend beyond the scope of passive sorption. Nevertheless, despite these insights, no study has directly investigated the co-exposure of dissolved cobalt (II) ions and MCN in *D. rerio* embryos or larvae using within an integrated framework encompassing developmental, functional, and biochemical endpoints. Consequently, the question remains unresolved as to whether MCN-mediated cobalt (II) sequestration yields true toxicological protection, provides no net biological benefit, or instead reshapes cobalt toxicity through nano–bio interactions during early vertebrate development.

In this study, we systematically evaluated the single and combined effects of dissolved Co (II) and MCN in *D. rerio* embryos and larvae. We employed an integrated multi-endpoint framework spanning survival, morphological development, locomotor behavior, and biochemical stress responses. The objective of the study was to test the hypothesis that physicochemical cobalt (II) sequestration by magnetite–chitosan nanocomposites (MCN) may provide biological protection. Specifically, we hypothesized that, through nano–bio interactions, MCN might mitigate cobalt-induced effects during early fish development. By directly contrasting Co, MCN and co-exposure (Co + MCN) conditions, we distinguished between sorption-driven mitigation and biologically mediated modulation of toxicity. The results provide insight into the assumption that chemical removal of metals necessarily translates into biological safety and contribute to refining environmental risk assessment of multifunctional nanomaterials.

## 2. Materials and Methods

### 2.1. Chemicals Used for Synthesis

Analytical grade or higher purity reagents were used in this study, the main for the synthesis were as follows: FeCl_2_·4H_2_O (CAS No. 13478-10-9) (Sigma Aldrich, Darmstadt, Germany), FeCl_3_ (CAS No. 7705-08-0) (Sigma Aldrich, Germany), Chitosan powder (100.000–300.000 m. w., Sigma Aldrich, Germany), Glutaraldehyde solution (50 wt.% in water) (Sigma Aldrich, Germany), NH_4_OH (CAS No. 1336-21-6, Honeywell, Fluka, Seelze, Belgium); NaOH (CAS No. 1310-73-2, Chempur, Piekary Śląskie, Poland), EtOH (CAS No. 64-17-5, Honeywell, Belgium).

### 2.2. Synthesis of Magnetite-Chitosan Nanocomposite (MCN)

The synthesis of fresh portion of MCN involved the preparation of magnetite nanoparticles via the co-precipitation method, followed by their immobilization into dissolved chitosan using a crosslinking agent as described in Šemčuk et al. [35]. Prepared solutions of FeCl_2_·4H_2_O and FeCl_3_ were combined following a 1:2 (Fe^2+^:Fe^3+^) ratio under argon atmosphere and mixed for 30 min. Then NH_4_OH was added dropwise until pH reached 9 with subsequently temperature raised to 75 °C. After 2 h intensive stirring the resulting Fe_3_O_4_ nanoparticles were separated, washed with ethanol and Milli-Q (Type I) water until a pH about 6, dried at 50 °C and homogenized for better dosage. The chitosan powder was dissolved in solution 1.5% acetic acid solution and mixed with water dispersed Fe_3_O_4_ nanoparticles in ratio 7:3 (*w*/*w*) to obtain 30 wt.% load of magnetite. After 2 h of intensive mixing the 1 wt.% of glutaraldehyde (wt./wt. chitosan) was added dropwise as a crosslinking agent. Then followed an additional 2 h of mixing at 50 °C, with a gradual increase in pH to 9 using an NaOH 0.5 M solution. Finally, the resulting MCN was collected using a magnet, washed with ethanol and Milli-Q (Type I) water, and dried and homogenized.

### 2.3. Characterization Techniques Used for MCN

The synthesized MCN was characterized using various techniques. The surface morphology was imaged using scanning electron microscopy (SEM) on Helios NanoLab 650 (FEI, Eindhoven, The Netherlands, 2011) and transmission electron microscopy (TEM) on Tecnai G2 F20 X-TWIN (FEI, The Netherlands). The XRD patterns were recorded with X-ray diffractometer Smart Lab (Rigaku, Tokyo, Japan). The Fourier transform infrared (FTIR) spectra were obtained using Alpha spectrometer (Bruker, Inc., Ettlingen, Germany) equipped with a Platinum diamond attenuated total reflectance accessory and a room-temperature RT-DLATGS detector (Bruker, Inc., Ettlingen, Germany). Mössbauer spectra were measured using a Mössbauer spectrometer (Wissenschaft Liche Elektronik GmbH, Starnberg, Germany) with ^57^Co (Rh) source. Magnetometer consisting of SR510 lock-in amplifier (Stanford Research Systems, Sunnyvale, CA, USA), FH-54 Gauss/Tesla meter (Magnet Physics, Cologne, Germany), and a laboratory magnet supplied by an SM 330-AR-22 power source (Delta Elektronika, Zierikzee, The Netherlands) was used to record magnetization curves.

### 2.4. Adsorption Capacity and Removal Efficiency

Batch sorption experiments were conducted in E3 solution with initial Co (II) concentrations ranging from 15.625 to 2000 µmol L^−1^ or equal from 0.92 to 117.87 mg L^−1^, achieved by dissolving the required amount of CoCl_2_·6H_2_O, and for MCN 1 g L^−1^. The selected concentrations are usual for such experiments and will facilitate the assessment of the sorption properties of the sorbents under study, as well in the event of an accident, similar or higher concentrations are likely. The adsorption experiments were performed in E3 medium with a pH of approximately 7.2, consistent with the zebrafish embryo exposure conditions. The pH was not artificially adjusted in order to maintain the same physicochemical environment used in the biological exposure experiments. The experiments were carried out at 15 °C with gentle shaking throughout the exposure period. After 24 h exposure, the MCN was separated using a neodymium magnet, and the equilibrium concentrations of Co (II) were determinate in the solutions with optical emission spectrometry using inductively coupled plasma (ICP-OES, Optima7000DV, Perkin Elmer, Waltham, MA, USA) at a wavelength of 228.616 nm. The measurement results were used to evaluate adsorption capacity at equilibrium (*Q_e_*) and removal efficiency (*RE*) of MCN sorbent for the sorption of Co (II) in E3 water samples using the equation for calculations:

(1) $$Q_{e}=\;\frac{\left(C_{0}-C_{e}\right)\cdot V}{m}$$

(2) $$RE=\frac{C_{0}-C_{e}}{C_{0}}\times 100\%$$

where *RE* (%)—removal efficiency of MCN, *C_0_* and *C_e_* (mg L^−1^)—represents the initial and final concentrations of Co (II) in the E3 solution, *Q_e_* (mg g^−1^)—adsorption capacity, *V* (L) is the volume of sample and *m* (g) is the mass of added MCN into the sample.

Afterwords, the obtained results were plotted as *RE* and *Q_e_* dependence on initial concentrations and using OriginPro 2025 Software (OriginLab Corporation, Northampton, MA, USA), respectively.

Adsorption capacity data was analyzed using Freundlich, Langmuir, and Temkin non-linear adsorption isotherms Equation (3)–(5):

(3) $$Q_{e}=K_{F}C_{e}^{1/n}$$

(4) $$Q_{e}=\frac{Q_{max}K_{L}C_{e}}{1+K_{L}C_{e}}$$

(5) $$Q_{e}=B\ln\left(A_{T}C_{e}\right)$$

(6) $$R_{L}=\frac{1}{1+{K_{L}C}_{0}\;}$$

where *Q_e_* (mg g^−1^)—adsorption capacity at equilibrium, *Ce* (mg L^−1^)—equilibrium concentration of Co^2+^ in solution, *K_F_*—Freundlich adsorption constant, *n*—Freundlich adsorption intensity parameter, *K_L_* (L mg^−1^)—Langmuir adsorption constant, *Q_max_* (mg g^−1^)—maximum adsorption capacity, *A_T_*—Temkin equilibrium binding constant, *B*—Temkin constant of adsorption.

Also, the separation factor of Langmuir isotherm *R_L_* (L mg^−1^) (Equation (6)) was calculated to indicate the adsorption type: unfavorable (*R_L_* > 1), linear (*R_L_* = 1), favorable (0 < *R_L_* < 1), or irreversible (*R_L_* = 0) [13]. The quality of the fit was assessed with correlation coefficients (*R^2^*) and chi-square test (*χ^2^*) (Equation (7)):

(7) $$\chi^{2}=\sum \frac{{\left(Q_{exp}\;-\;Q_{calc}\right)}^{2}}{Q_{calc}}$$

where *Q_exp_* is the experimentally determined adsorption capacity and *Q_calc_* is the adsorption capacity predicted by the model.

### 2.5. Chemicals and Exposure Conditions

Cobalt was administered as cobalt (II) chloride hexahydrate (CoCl_2_·6H_2_O; CAS No. 7791-13-1; Sigma-Aldrich, Germany). Stock solutions were prepared in an E3 medium and diluted to the required exposure concentration immediately prior to use.

Prior to exposure, MCNs were suspended in E3 medium and sonicated (5 min) to ensure homogenous dispersion.

Fertilized *Danio rerio* (Hamilton, 1822) embryos were randomly distributed into four treatment groups: control (E3 medium only); Co (0.1 g L^−1^ Co (II), introduced by dissolving cobalt (II) chloride hexahydrate, CoCl_2_·6H_2_O); MCN (1 g L^−1^ magnetite–chitosan nanocomposite); and Co + MCN (combined exposure to 0.1 g L^−1^ Co (II) and 1 g L^−1^ MCN). The cobalt (II) concentration was selected to induce measurable biological responses [36] while remaining within the previously determined adsorption capacity range of MCN. The MCN concentration of 1 g L^−1^ was selected as a standard working concentration based on preliminary adsorption experiments and was kept constant across treatments to ensure comparability between single and combined exposures.

### 2.6. Zebrafish Maintenance and Breeding

Adult wild-type AB strain *Danio rerio* were maintained in a ZebTec Active Blue recirculating aquatic system (Tecniplast, Buguggiate, Italy) under standard laboratory conditions: temperature 28 ± 1 °C, pH 7.5 ± 0.2, conductivity 600 ± 20 µS cm^−1^, and a 14:10 h light/dark photoperiod (lights on at 07:00). Water was UV-sterilized and filtered through mechanical, chemical (activated carbon), and biological filtration units. Ammonia, nitrite, and nitrate levels were maintained within recommended ranges (ammonia < 0.01 mg L^−1^, nitrite < 0.1 mg L^−1^, nitrate < 25 mg L^−1^).

Fish were fed four times daily with a commercial microencapsulated dry feed (Zebrafeed, Sparos Lda, Olhão, Portugal; particle size 400–600 µm), supplemented twice daily with live *Artemia* nauplii (INVE Aquaculture, Dendermonde, Belgium).

Spawning was induced using male-to-female ratios of 2:1 in breeding tanks equipped with mesh-bottom inserts to prevent egg predation. Breeding was synchronized by light onset, and fertilized eggs were collected within 3 h post-fertilization (hpf). Embryos were rinsed with sterile E3 medium (5.0 mM NaCl, 0.17 mM KCl, 0.33 mM CaCl_2_, 0.33 mM MgSO_4_, pH 7.2) and inspected under a stereomicroscope (Meiji Techno RZ, Meiji Techno Co., Ltd., Saitama, Japan) for viability and developmental stage. Only embryos at the 2–4 cell stage and free of visible abnormalities were selected for experimental use.

All experimental procedures involving embryos were conducted in accordance with OECD Test Guideline 236 (Fish Embryo Acute Toxicity Test, FET) [37] and adhered to European Directive 2010/63/EU [38] on the protection of animals used for scientific purposes and were approved by the Nature Research Center Animal Welfare Council (2025-01-28 No. GGT10).

For bioassays, exposures were performed in 90 mm glass Petri dishes, with 30 embryos or larvae per dish in 30 mL of test solution. Each treatment condition was conducted in triplicate, with three independent biological replicates.

All exposures were performed under static, non-renewal conditions at 28 ± 0.5 °C, with a 14:10 h light/dark photoperiod consistent with maintenance conditions. Embryos were monitored daily for survival. Embryos or larvae were considered dead if they displayed absence of heartbeat, lack of somite formation, coagulation, or failure to develop a visible tail detachment. Dead individuals were promptly removed to prevent decomposition or contamination of the exposure medium. The cumulative percentage of hatched larvae was calculated for each treatment group at each time point (48, 72, and 96 h post-exposure).

### 2.7. Tail Activity Assessment (24 h Post-Exposure)

To evaluate early neuro-motor function, tail activity of embryos was assessed at 24 h post-exposure. Embryos from each treatment group were transferred onto a concave object slide containing a droplet of tested medium and placed under a microscope (Optika B-600, Optika S.r.l., Ponteranica, Italy). The droplet volume (~50 µL) was sufficient to fully submerge the embryo while gently restricting movement within the field of view. The embryo was recorded for 2 min using a digital video capture system integrated with the microscope. Videos were analyzed using DanioScope™ software 1.2 (Noldus Information Technology B.V., The Netherlands), which automatically detects spontaneous tail flicks and quantifies temporal movement patterns. Two key behavioral parameters of embryos were extracted: burst activity/inactivity defined as the percentage of time the embryo’s tail was actively moving (bursting) versus inactive; burst count per minute the number of discrete tail movements per minute, representing overall excitability or motor responsiveness.

### 2.8. Heart Rate Assessment 48–96 h Post-Exposure

Heart rate (HR) was assessed at 48, 72, and 96 h post-exposure as a non-invasive indicator of developmental and cardiophysiological status. At each timepoint, a subset of live embryos or larvae (*n* = 15 per treatment group) was randomly selected for analysis. Each individual was gently transferred using a Pasteur pipette into a small droplet (~50 µL) of the corresponding test solution, placed in the depression of a concave microscope slide to gently restrict movement without the use of anesthesia. This setup enabled clear visualization of the cardiac region while maintaining physiological conditions. Heart activity was recorded under a stereomicroscope (Meiji Techno RZ, Meiji Techno Co., Ltd., Saitama, Japan) using slow-motion video capture. Heartbeats were counted over a 10 s video segment and converted to beats per minute by multiplying the count by six. This approach provided enhanced temporal resolution and ensured accurate quantification of heart rate. Only viable embryos or larvae displaying clear and rhythmic cardiac contractions were included in the final analysis.

### 2.9. Locomotor Behavior (96 h Post-Exposure)

At 96 h, larval locomotor activity was evaluated to assess potential neurofunctional effects of tested materials. Behavioral testing was performed using the DanioVision™ Observation Chamber (Noldus Information Technology B.V., Wageningen, The Netherlands) integrated with EthoVision^®^ XT software (version 17) for automated video tracking and behavioral analysis. Larvae were individually transferred into a 48-well clear-bottom polystyrene plate (one larva per well; ~200 µL per well of the corresponding exposure solution). Each well was defined as an independent behavioral arena within the software. The chamber was maintained at 28 ± 0.5 °C, and recordings were performed under controlled conditions using infrared backlighting to prevent visual stimulation during dark phases. The DanioVision system was equipped with a GigE (Basler AG, Ahrensburg, Germany) infrared-sensitive digital camera operating at 30 frames per second (fps) with 1080p resolution, providing high temporal and spatial resolution for movement detection. Prior to testing, larvae were visually screened, and only morphologically normal and freely swimming individuals were included. Each trial consisted of three standardized phases: acclimation phase (10 min)—constant light to allow behavioral stabilization; photomotor phase: alternating 10 min of light and 10 min of darkness, assessing light-modulated spontaneous locomotion; and stimulus-evoked phase: a double mechanical tap was applied to the plate using the chamber’s integrated stimulus device; movement was recorded for 10 s before and after the stimulus to evaluate startle responses.

EthoVision XT software 17.0 automatically differentiated larvae from the background using contrast-based segmentation and tracked their positions throughout the trial. Tracking parameters were optimized for zebrafish larvae, with a movement threshold set at 0.2 mm/s to distinguish active movement from drift or noise. A smoothing filter (5-frame sliding average) was applied to reduce motion artifacts.

The following behavioral endpoints were extracted: total distance moved (mm per 10 s bin) over the full session; phase-averaged locomotor activity, calculated separately for light and dark phases; and tap-evoked response, expressed as the difference in distance moved between the 10 s post-stimulus and pre-stimulus (Δ movement). Each treatment group included 16 larvae, tested in two independent trials.

### 2.10. Assessment of Oxidative Stress–Related Biomarkers

After 96 h of exposure, larvae were collected, rinsed with ice-cold phosphate-buffered saline (PBS), pooled (*n* = 10 per sample), and homogenized on ice in 500 µL PBS (pH 7.4). Homogenates were centrifuged at 10,000× *g* for 15 min at 4 °C, and supernatants were collected for analysis.

The activity of superoxide dismutase (SOD) was measured using a colorimetric assay kit (Thermo Fisher Scientific, Frederick, MD, USA, Cat. No. EIASODC; 450 nm). The extent of lipid peroxidation was determined by measuring the levels of malondialdehyde (MDA) using a TBARS kit (Thermo Fisher Scientific, Cat. No. EEA015; 532 nm). The quantification of hydrogen peroxide (H_2_O_2_) was performed using a peroxide assay kit (Sigma-Aldrich, Cat. No. MAK311; 585 nm). Total protein concentration was determined by the bicinchoninic acid (BCA) method (Thermo Fisher Scientific) with bovine serum albumin standards (562 nm). Protein concentrations (mg mL^−1^) were calculated from the standard curve, and all biochemical parameters were normalized to total protein content.

### 2.11. Statistical Analysis

All statistical analyses were performed using STATISTICA 10.0 (TIBCO Software Inc., Palo Alto, CA, USA) and GraphPad Prism 11 (GraphPad Software, Boston, MA, USA). Prior to analysis, all datasets were examined for compliance with statistical assumptions: normality was assessed using both the Kolmogorov–Smirnov and Shapiro–Wilk tests. Homogeneity of variances was tested using Levene’s test. Where assumptions were met, parametric tests were applied; otherwise, equivalent non-parametric tests were used. For single-factor comparisons, data were analyzed using one-way ANOVA followed by Tukey’s Honest Significant Difference (HSD) post hoc test to identify specific pairwise differences. Tukey’s HSD controls for the family-wise error rate and was applied only when ANOVA indicated a statistically significant overall effect (*p* < 0.05). If ANOVA assumptions were violated, Kruskal–Wallis tests followed by Dunn’s post hoc test were applied. For phase-dependent locomotor activity during light/dark transitions, a repeated-measures ANOVA or generalized linear mixed model (GLMM) was used.

## 3. Results and Discussion

The Safe-by-Design (SbD) framework aims to integrate safety considerations into the development of nanomaterials and nano-enabled products from the earliest stages of design, alongside functionality and technical performance [39]. In the context of nanomaterials proposed for environmental remediation, this approach requires moving beyond demonstrations of chemical effectiveness based solely on sorption capacity or removal efficiency and instead evaluating potential biological impacts under realistic and combined exposure scenarios [40,41]. Here, we implement a SbD framework to MCN by integrating sorption performance with biological effect assessment under single and combined exposure conditions. This approach critically tests whether physicochemical cobalt (II) ions immobilization by MCN translates into tangible developmental protection or instead reveals unanticipated biological effects not captured by conventional abiotic performance metrics.

### 3.1. Characterization of MCN

#### 3.1.1. Electron Microscopy (SEM, TEM)

**Figure 1:**
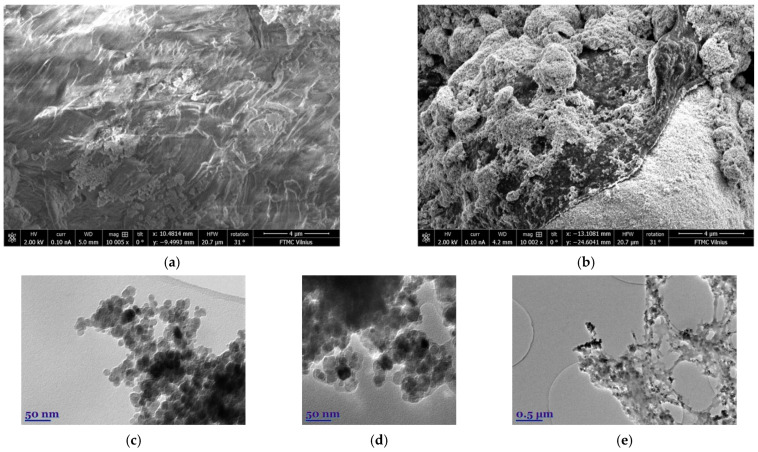
SEM images of pure chitosan (**a**), MCN (**b**) and TEM images of synthesized magnetite (**c**), MCN (**d**,**e**).

The comparison images taken with SEM of pure chitosan and synthesized MCN are shown in Figure 1a,b. It is clearly seen that after modification the surface becomes more rustling or porous like, due to attraction of magnetite nanoparticles that seem to be distributed almost evenly over the entire surface. The more detailed analysis of the magnetite (c) and MCN (d, e) surface was achieved using TEM technique. The synthesized Fe_3_O_4_ nanoparticles (c) appeared as agglomerate of 16–20 nm spheric nanoparticles without any impurities. In approximation view of MCN (d) confirmed same sized to (c) magnetite nanoparticles are attached on surface as alone and as small agglomerates. Moreover, high scaling image of MCN (e) shows that it is micro sized nanocomposite with heterogenic structure decorated with nanoparticles of magnetite.

#### 3.1.2. X-Ray Diffraction (XRD)

**Figure 2:**
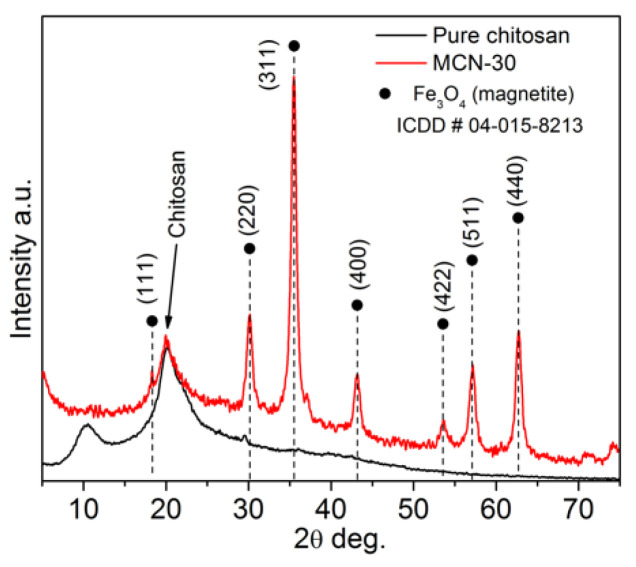
XRD patterns of chitosan and MCN, with typical Fe_3_O_4_ diffraction peaks.

The XRD patterns recorded for chitosan and MCN in the 2θ range from 5° to 75° are presented in Figure 2. The characteristic wide diffraction peak observed at approximately 20° belongs to chitosan and appears in same position in the synthesized MCN. As expected, the characteristic diffraction peaks for Fe_3_O_4_ (ICDD card no. #00-019-0629) at the (111), (220), (311), (400), (422), (511), and (440) crystal planes were registered in the MCN pattern. These results are consistent with those of a previous study [35], suggesting the same trend that crystal structure of magnetite remains unchanged during the synthesis of MCN, thus preserving its structural and magnetic properties.

#### 3.1.3. Fourier Transform Infrared Spectroscopy (ATR-FTIR)

**Figure 3:**
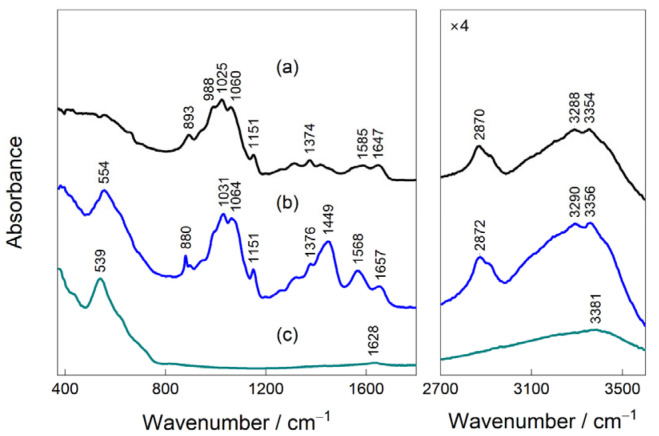
ATR-FTIR spectra of magnetite (**a**), chitosan (**b**), MCN (**c**) in 400–1750 cm^−1^ and 2700–3600 cm^−1^ spectral regions.

Figure 3 presents ATR-FTIR spectra of chitosan powder, MCN, and magnetite. The spectrum of pristine chitosan is dominated in the fingerprint region by intense bands at ~1020–1080 cm^−1^, attributed to C–O–C and C–O stretching vibrations of the polysaccharide backbone, together with a band near 893 cm^−1^ associated with saccharide ring vibrations [42,43,44,45]. The bands observed near ~1650 and 1568–1585 cm^−1^ correspond to residual amide I (νC=O) and amide II (νC–N + δNH) vibrations, consistent with partially deacetylated chitosan. In the higher-wavenumber region, the broad absorption between 3200 and 3500 cm^−1^ arises from overlapping O–H and N–H stretching modes, reflecting the extensive hydrogen-bonding network typical of chitosan. The spectrum of magnetite is characterized by a strong absorption centered at 539 cm^−1^, which is assigned to Fe–O stretching vibrations in the inverse spinel lattice [46]. The absence of significant bands in the 2700–3600 cm^−1^ region confirms the inorganic nature of magnetite, while the peak centered at 3381 cm^−1^ corresponds to surface hydroxyl groups on the iron oxide lattice or adsorbed atmospheric water.

MCN exhibits spectral features originating from chitosan, magnetite, and glutaraldehyde, confirming successful formation of the hybrid material. The Fe–O band remains clearly visible in the low-wavenumber region, and due to interaction with chitosan, is shifted by 15 cm^−1^ to higher frequencies. These changes point to interfacial interactions between magnetite surfaces and chitosan functional groups, most plausibly involving coordination or hydrogen bonding through –NH_2_ and –OH moieties. The characteristic chitosan bands in the 1020–1080 cm^−1^ range are retained, indicating preservation of the polysaccharide backbone. However, stark spectral changes appear in the 1200–1700 cm^−1^ range, mostly related to crosslinking of chitosan with glutaraldehyde [47,48]. Since the ~1720 cm^−1^ range is free from spectral bands, glutaraldehyde is completely consumed in crosslinking with chitosan. Additionally, spectrograms of 2 mM and 0.25 mM of Co (II) dissolved in an E3 solution were recorded before and after adsorption using a UV-Vis spectrometer (see Supplementary Figure S1). The results show the absence of characteristics Co (III) peaks at 340 and 460–470 nm, as well as no absorption peaks in the 600–700 nm range, which are characteristic of tetrahedral [CoCl_4_]^2−^ complexes. Furthermore, the absence of Co(OH)_2_ precipitation in all tested solutions confirms that the CoCl_2_·6H_2_O salt was fully dissolved. In the solution, cobalt remains in its divalent state as Co (II), predominantly in the form of [Co(H_2_O)_6_]^2+^, as evidenced by the weak absorption peaks around 510 nm, which are of low intensity due to relatively low cobalt concentrations. Moreover, measurements of Co (II) concentrations in the solutions used during desorption and batch sorption experiments, as determined by ICP-OES at 228.6 nm (characteristic wavelength for Co (II) ions) further confirm this observation.

#### 3.1.4. Mössbauer Spectroscopy

**Figure 4:**
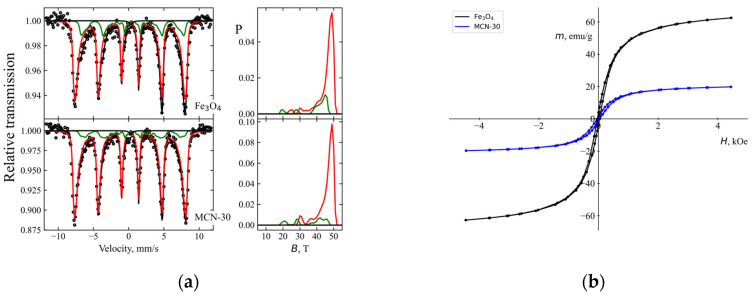
Mössbauer spectra of (**a**) synthesized magnetite nanoparticles and the MCN (30 wt.% Fe_3_O_4_) composite, including the corresponding hyperfine field distributions (right); (**b**) magnetization curves of the same samples.

Mössbauer spectra of synthesized magnetite and MCN (Figure 4) were fitted using two hyperfine field distributions with distinct isomer shifts (Table 1). These shifts are characteristic of the tetrahedral A (Fe^3+^) and octahedral B (Fe^3+^ and Fe^2+^) sublattices of magnetite. The first distribution also accounts for maghemite (γ-Fe_2_O_3_), where only Fe^3+^ is present. The spectral area ratio of the two distributions indicates a relative Fe^2+^ content of *I_Fe_*^2+^ = 0.5*I_B_*/(*I_A_* +* I_B_*) = 0.05–0.09, which is typical of highly non-stoichiometric magnetite, suggesting a composition close to maghemite. The average size of the nanoparticles (16–18 nm) was determined using Equation (8) given in [49]:

(8) $$\langle B\rangle =B_{0}\left(1-\frac{kT}{2KV}\right)$$

This calculation accounts for a ≈7–10% decrease in the average hyperfine field ⟨B〉 of the dominant distribution relative to that of the bulk magnetite A sublattice (*B_A_* ≈ 49 T) or bulk maghemite (49.1; 50.3 T) [50], resulting from the reduced particle volume V. Here, K represents the magnetic anisotropy of magnetite and k is the Boltzmann constant. An additional decrease in the relative area of the second subspectrum (sublattice B) in the magnetite–chitosan composites indicates further oxidation of the nanoparticles.

### 3.2. Removal Efficiency and Adsorption Capacity Study

**Figure 5:**
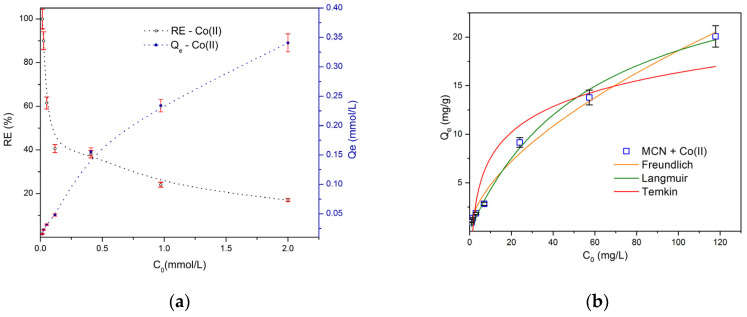
Dependence of adsorption capacity and removal efficiency (**a**) on initial concentration of Co (II) (mmol L^−1^) using MCN in E3 solution, and adsorption capacity data analysis (**b**) using Freundlich, Langmuir, Temkin adsorption isothermal non-linear fit.

The removal efficiency (*RE*, Equation (2)) and adsorption capacity (*Q_e_*, Equation (1)) of MCN for Co (II) in E3 solution was calculated, and the results were plotted as a function of initial concentration *C*_0_ vs. *RE*, *Q_e_* (Figure 5a). The maximum experimental adsorption capacity was determined to be 0.34 mmol g^−1^ (20.08 mg g^−1^) at initial Co (II) concentration of 2 mmol L^−1^. While removal efficiency (*RE*) of MCN at initially very low concentrations of Co (II) tended to be 100%, at concentrations from 0.05 to 0.5 mmol L^−1^ it varied from 80% to 40%, respectfully, and at initial concentrations higher than 1 mmol L^−1^ it decreased to 17% at 2 mmol L^−1^. To evaluate the adsorption mechanism, an analysis of these data was performed using a non-linear approximation of the Freundlich, Langmuir, and Temkin adsorption isotherm models (Figure 5b). The highest correlation coefficient (*R*^2^ = 0.993) and the lowest chi-square test value (*χ*^2^ = 0.393) were provided for Langmuir model compared to other (Table 2). All this suggests a favorable monolayer adsorption of Co (II) onto a functionally homogenous surface of MCN with theoretically calculated maximum adsorption capacity (*Q_max_*) of 29.553 mg g^−1^, which is in good agreement with the experimental observations. The predominance of the Langmuir model indicates that adsorption occurs mainly through monolayer binding of Co (II) ions on energetically similar active sites of the MCN surface. This behavior is consistent with the presence of amino (–NH_2_) and hydroxyl (–OH) functional groups in the chitosan matrix, which are known to form coordination interactions with metal ions. The calculated separation factor *R_L_* = 0.333 (Equation (6)) met the criteria 0 < *R_L_* < 1 that confirms the favorable adsorption of Co (II) onto MCN. In addition, the magnetic Fe_3_O_4_ nanoparticles provide a highly reactive surface that can further contribute to metal ion binding that is also indicated by high correlation (*R*^2^ = 0.989) of Freundlich model that best describes a multilayer adsorption on heterogeneous surface. Similar adsorption behavior has been reported for other chitosan-based magnetic nanocomposites used for heavy metal removal from aqueous systems [51,52]. Additionally, to demonstrate the reusability of the adsorbent, sorption–desorption experiments were performed using the same amount of MCN-30 in E3 solution with Co (II), followed by desorption using HNO_3_ solutions (Supplementary Figure S2). The desorption studies confirmed the reusability of MCN-30.

### 3.3. Mortality and Hatching Success

**Figure 6:**
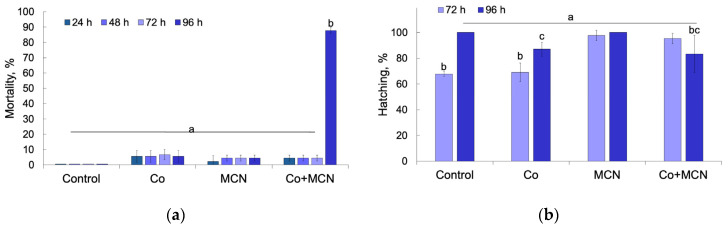
Developmental toxicity of Co, MCN, and their combined exposure (Co + MCN) in *Danio rerio* embryos. (**a**) Cumulative mortality (%) recorded at 24, 48, 72, and 96 h post-exposure. (**b**) Hatching success (%) assessed at 72 and 96 h post-exposure. Data are presented as mean ± SD. Different letters above bars indicate statistically significant differences among treatments at the same time point (*p* < 0.05).

Previous studies indicate that cobalt toxicity in fish occurs across a broad concentration range and may affect multiple biological systems depending on exposure level and developmental stage. For example, Reinardy et al. [36] reported a 96 h LC_50_ of 35.3 ± 1.1 mg L^−1^ for larval zebrafish and demonstrated that chronic exposure to 15–25 mg L^−1^ Co (II) induced DNA damage in sperm and reduced reproductive success in adult zebrafish. Developmental toxicity studies using zebrafish embryo–larval assays have shown that exposure to 10–100 mg L^−1^ Co (II) can impair hatching, induce bradycardia, and cause morphological abnormalities such as yolk sac and pericardial edema [53]. Experiments examining metal effects on zebrafish eggs and larvae further demonstrate strong concentration dependence, with exposures up to 500 mg L^−1^ causing complete mortality within 96 h [54]. In other fish species, short-term exposure to 50–300 mg L^−1^ CoCl_2_ has been associated with pronounced hematological disturbances and erythrocyte abnormalities, confirming the toxicological impact of elevated cobalt concentrations [55]. At the physiological level, cobalt (II) can interfere with sensory systems; for instance, treatment with millimolar concentrations of CoCl_2_ has been shown to block calcium transport in lateral line hair cells of zebrafish larvae, demonstrating the sensitivity of mechanosensory pathways to cobalt (II) exposure [56]. In contrast to waterborne toxicity, dietary cobalt supplied at trace levels (approximately 0.19–0.28 mg kg^−1^ feed) has been reported to enhance antioxidant capacity, growth performance, and immune responses in fish, indicating that cobalt may exert beneficial physiological effects when present at low nutritional levels [57]. Environmental risk assessments further show that chronic toxicity thresholds for aquatic organisms can occur at very low concentrations, with chronic EC_10_ values ranging from approximately 1.23 µg L^−1^ to 31,800 µg L^−1^ across marine species, highlighting large interspecies variability in cobalt (II) sensitivity [18]. In present study, cumulative mortality of *D. rerio* embryos and larvae was significantly affected by exposure time, treatment, and their interaction (two-way ANOVA; time: F_3.32_ = 218.5, *p* < 0.001; treatment: F_3.32_ = 253.8, *p* < 0.001; time × treatment: F_9,32_ = 215.9, *p* < 0.001), demonstrating a strong time-dependent modulation of toxicity across exposure scenarios (Figure 6a). Mortality remained low and comparable among the control, Co, and MCN groups throughout the first 72 h of exposure, indicating limited acute lethality under individual treatments. In contrast, a pronounced and abrupt increase in mortality was observed exclusively in the combined Co + MCN group at 96 h, reaching approximately 85–90% and differing significantly from all other treatments (Tukey HSD, *p* < 0.001). This delayed but severe lethality indicates a non-additive interaction between cobalt (II) ions and MCN that emerges only at later developmental stages and cannot be explained by cumulative toxicity of either stressor alone.

Hatching success was likewise influenced by exposure time, treatment, and their interaction (two-way ANOVA; time: F_1,16_ = 15.05, *p* = 0.0013; treatment: F_3,16_ = 11.50, *p* = 0.0029; time × treatment: F_3,16_ = 13.47, *p* = 0.0012), revealing a contrasting response pattern relative to mortality (Figure 6b). At 72 h, Co exposure significantly reduced hatching compared to the control, whereas single MCN exhibited hatching rates comparable to, or slightly higher than, those of the control, suggesting that MCN did not impair early developmental processes required for successful chorion rupture. In the combined Co + MCN treatment, hatching was partially restored relative to group with Co, indicating that MCN may reduce effective cobalt (II) ions interference during early embryogenesis, potentially through reduced free Co (II) availability or altered interaction with the chorion. However, this apparent early-stage mitigation did not translate into improved survival, as evidenced by the dramatic late-stage mortality observed at 96 h.

The divergence between hatching and survival responses highlights a decoupling between early developmental compensation and later organism-level viability under combined exposure. While MCN co-exposure may transiently alleviate cobalt-induced developmental delay, it simultaneously appears to predispose larvae to severe late-stage toxicity. This pattern suggests that nanomaterial–metal interactions reshape cobalt toxicity in a stage-specific manner, potentially through altered cobalt distribution, nanoparticle-associated transport, or amplification of oxidative and physiological stress during post-hatching transitions characterized by increased metabolic demand and organ maturation [58].

### 3.4. Early Embryonic Behavior

**Figure 7:**
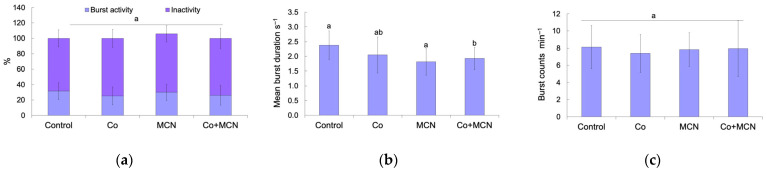
Behavioral performance of *Danio rerio* embryos at 24 h post-exposure to Co, MCN and their combination (Co + MCN). The assessed endpoints include locomotor states (**a**), mean burst duration (**b**), and burst frequency (**c**). Different letters indicate statistically significant differences among groups (*p* < 0.05, mean ± SD).

At 24 h post-exposure, behavioral endpoints were assessed to evaluate potential neurotoxic effects of Co, MCN, and their combination (Co + MCN) on *D. rerio* embryos. As shown in Figure 7a, no statistically significant differences were observed in the proportion of burst activity versus inactivity across the treatment groups. A Kruskal–Wallis test revealed no overall treatment effect (H (3, *n* = 71) = 6.49, *p* = 0.090), and pairwise comparisons confirmed the absence of significant differences (all *p* > 0.05). In contrast, a significant effect was observed in the mean burst duration (Figure 7b). One-way ANOVA indicated a significant overall treatment effect (F_3,67_ = 5.33, *p* = 0.002). Post hoc Tukey’s HSD test identified the Co + MCN group as significantly different from both the control (*p* = 0.026) and MCN group (*p* = 0.007), exhibiting a marked reduction in burst duration. The mean burst durations were 2.48 s (control), 2.05 s (Co), 2.41 s (MCN), and 1.93 s (Co + MCN).

This selective reduction in burst duration, despite preserved movement initiation, highlights a mechanistically informative dissociation between motor activation and sustained output. Such patterns have been increasingly recognized in developmental neurotoxicology. For example, Zakaria et al. (2022) [59] reported that surface-modified gold nanorods impaired zebrafish locomotor coordination without affecting movement frequency. Refs. [60,61] described nanoparticle-driven disruption of behavioral and endocrine endpoints without overt suppression of activity. Our results extend this pattern to cobalt–nanoparticle co-exposure: the Co + MCN group exhibited a significant reduction in burst duration, clustering separately from all other treatments. This synergistic effect may reflect enhanced Co (II) uptake or redistribution facilitated by magnetite–chitosan nanoparticles, consistent with hypothesis that nanoparticles modulate metal ion bioavailability and exacerbate neuromotor toxicity through ROS generation, mitochondrial dysfunction, or altered neurotransmitter signaling [62,63,64]. Given that burst duration correlates with neuromuscular coordination and central pattern generator output, these findings suggest compound-level interference with early neural circuits responsible for patterned motor behavior.

No significant differences were found in burst frequency across groups (Figure 7c). One-way ANOVA did not detect treatment effect (F_3,67_ = 0.623, *p* = 0.603), and Tukey’s HSD tests confirmed the absence of statistically significant pairwise differences (all *p* > 0.6). These results indicate that the rate of locomotor burst initiation was unaffected by any of the tested conditions within the short-term (24 h) exposure window.

### 3.5. Heart Rate Alteration

Heart rate (HR) is a sensitive physiological biomarker reflecting early cardiac and systemic function during zebrafish development. HR was recorded at 48, 72, and 96 h post-exposure, revealing time-dependent and compound-specific effects.

**Figure 8:**
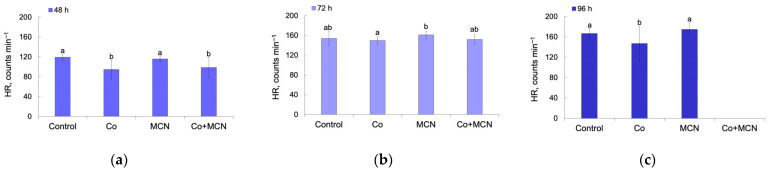
Heart rate (HR) of *Danio rerio* embryos and larvae at 48 h (**a**), 72 h (**b**), and 96 h (**c**) post-fertilization following exposure to Co, MCN and their combination (Co + MCN). Different letters indicate statistically significant differences among groups (*p* < 0.05, mean ± SD). Note: at 96 h post-exposure, all larvae in the Co + MCN group were dead (>85% mortality); thus, this group is excluded from statistical analysis and not shown in the graph.

At 48 h post-exposure, significant differences in HR were observed among treatments (one-way ANOVA: F_3,56_ = 10.46, *p* < 0.0001; Figure 8a). Embryos exposed to Co exhibited significantly reduced HR compared to controls (*p* < 0.001), consistent with early-stage bradycardia seen in metal exposed zebrafish [53,65]. A similar reduction was observed in the Co + MCN group relative to control (*p* = 0.002) and the MCN group (*p* = 0.013), while MCN alone did not affect HR (*p* = 0.917). These results demonstrate that Co-alone is the primary driver of early cardiotoxic effects, and that co-exposure with MCN does not attenuate, but rather maintains, this negative impact.

At 72 h post-exposure, HR differences remained significant although less pronounced (F_3,56_ = 3.42, *p* = 0.023; Figure 8b). Embryos exposed to MCN exhibited higher HR compared to Co (*p* = 0.020), suggesting a possible compensatory or stress response following nanoparticle exposure [60,66]. No other pairwise comparisons reached statistical significance.

At 96 h post-exposure, heart rate analysis could not be performed for the Co + MCN group due to >85% mortality, indicating severe and lethal toxicity of the combined exposure. Consequently, statistical analysis therefore included only the control, single Co, and MCN groups. A significant treatment effect was detected (F_2,42_ = 6.84, *p* = 0.003; Figure 7c). Larvae exposed to Co continued to exhibit bradycardia relative to control (*p* = 0.038), confirming persistent cardio depression. In contrast, MCN-exposed larvae displayed elevated HR compared with both the control (*p* = 0.038) and Co groups (*p* = 0.002), consistent with late-stage stress or compensatory tachycardia.

The complete mortality observed in the Co + MCN group by 96 h post-exposure provides compelling evidence of synergistic toxicity, far exceeding the effects of either exposure alone. Such outcomes may reflect enhanced cobalt (II) bioavailability or nanoparticle-facilitated cellular uptake disrupting cardiac ion homeostasis, redox regulation, or mitochondrial integrity, mechanisms supported by studies linking engineered nanoparticle metal exposures with amplified toxicity in early life stages [67,68].

### 3.6. Locomotor Behavior

**Figure 9:**
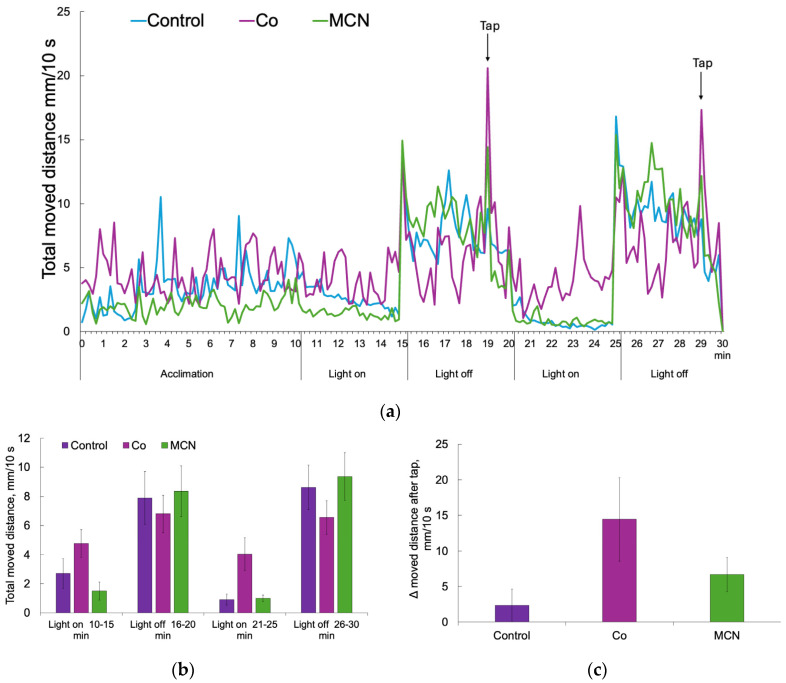
Locomotor behavior of *Danio rerio* larvae at 96 h post-exposure following exposure to Co or MCN groups. (**a**) Time-resolved total distance traveled (mm per 10 s) by control, Co, and MCN groups larvae during acclimation, alternating light and dark phases, and a mechanical tap stimulus. (**b**) Phase-averaged total distance traveled during light and dark phases (mean ± SE). (**c**) Tap-evoked locomotor response expressed as the change in distance traveled during the first 10 s after stimulation relative to the pre-stimulus baseline (mean ± SE).

At 96 h post-exposure, larval locomotor behavior was assessed to examine potential neurofunctional effects of Co and MCN groups, using time-resolved activity tracking, phase-averaged locomotion, and stimulus-evoked responses (Figure 9). Such multi-endpoint behavioral paradigms are widely applied and standardized for zebrafish larvae to detect subtle neurofunctional alterations that may not be evident from morphology alone [69,70]. Figure 9a illustrates the temporal dynamics of total distance moved by larvae in control, Co, and MCN groups during the recording session, which consisted of an initial acclimation period followed by alternating light-on and light-off phases and a double mechanical tap stimulus. During acclimation, locomotor activity gradually stabilized across all three groups, indicating comparable adaptation to the testing arena and excluding treatment-specific disturbances in baseline exploratory behavior. Throughout the recording, larvae in the control, Co, and MCN groups exhibited a characteristic photomotor response, characterized by reduced activity during light on phases and increased swimming during light off phases. This light–dark modulation of locomotion is a robust and conserved behavioral feature in zebrafish larvae, reflecting intact visual processing and motor output pathways [69,71]. The comparable alternating locomotor patterns observed across treatments indicate that exposure did not measurably disrupt visually driven regulation of spontaneous swimming behavior. Phase-averaged analysis of locomotor activity (Figure 8b) confirmed light condition as the primary determinant of swimming behavior. Across the control, Co, and MCN groups, total distance moved was consistently greater during light-off phases than during light-on phases, indicating intact photomotor regulation. Statistical analysis revealed a highly significant effect of light phase (χ^2^ = 56.81, *p* < 0.001), whereas no significant main effect of treatment was detected (χ^2^ = 0.68, *p* = 0.71), nor was a significant treatment × light interaction observed (χ^2^ = 10.25, *p* = 0.115). These results indicate that exposure in Co or MCN groups did not significantly modify the magnitude of light-driven locomotor activity. Functionally, this suggests that core sensory processing and motor output circuits remain intact and resilient to the applied exposures at the larval stage. In contrast to spontaneous locomotor activity, the acute response to mechanical stimulation revealed greater variability among treatment groups (Figure 9c). Tap-evoked locomotor responses, quantified as the change in distance traveled during the first 10 s following stimulation relative to the immediately preceding baseline, differed numerically across groups. Larvae exposed to Co showed the highest mean tap-evoked response, MCN-treated larvae displayed an intermediate response, and control larvae exhibited the lowest mean change. However, substantial inter-individual variability was observed within each group, including both positive and negative Δ values, reflecting heterogeneous startle-related behaviors such as rapid escape swimming and transient freezing. One-way ANOVA did not identify a statistically significant treatment effect (F = 2.27, *p* = 0.115), indicating that group-level differences in tap responsiveness could not be resolved under the present experimental conditions.

The dissociation between preserved spontaneous locomotor regulation and variable stimulus evoked responses suggests that baseline activity metrics are relatively insensitive to subtle neurofunctional modulation, whereas startle related endpoints may better capture early or heterogeneous alterations in sensorimotor responsiveness. Similar observations have been reported in studies showing that acute chemical exposure can preferentially affect startle or stress related behaviors without altering spontaneous swimming patterns [72,73]. Although group level differences in tap evoked response magnitude were observed in the present study, they did not reach statistical significance, indicating that any neurobehavioral effects in Co or MCN groups at 96 h post-exposure are subtle and variable rather than robustly expressed at the population level under the tested concentrations and exposure conditions.

### 3.7. Oxidative Stress Biomarkers

**Figure 10:**
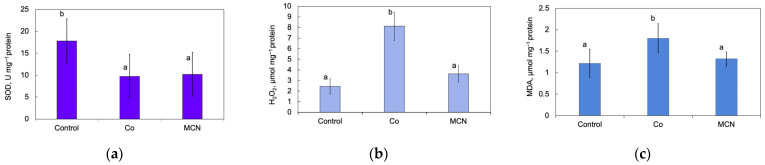
Antioxidant enzyme activity and oxidative stress markers in fish larvae following exposure to Co and MCN groups. (**a**) Superoxide dismutase (SOD) activity, (**b**) hydrogen peroxide (H_2_O_2_) concentration, and (**c**) malondialdehyde (MDA) levels. Values are presented as mean ± SD. Different letters indicate statistically significant differences among treatment groups (*p* < 0.05).

The assessment of oxidative stress biomarkers was conducted to evaluate redox imbalance and antioxidant defense responses in fish larvae following exposure to Co and MCN groups. Treatment-dependent effects were observed for superoxide dismutase (SOD) activity, hydrogen peroxide (H_2_O_2_) levels, and malondialdehyde (MDA) content (Figure 10). Owing to the extensive mortality (>85%) in the Co + MCN group at 96 h post-exposure, biochemical assessment of oxidative stress biomarkers was not feasible.

SOD activity exhibited significant variation among treatments (one-way ANOVA: F_2,15_ = 11.528, *p* < 0.001; Figure 10a). Larvae exposed to Co showed a marked reduction in SOD activity compared with the control group (*p* = 0.003), indicating impairment or depletion of enzymatic antioxidant defenses under metal-induced oxidative stress. A comparable decline in SOD activity was also observed in the MCN group relative to the control. SOD represents the primary enzymatic defense against ROS, catalyzing the conversion of superoxide radicals into hydrogen peroxide and oxygen [74]. Therefore, the reduction in SOD activity observed in the MCN group suggests that exposure to MCN altered the basal antioxidant defense capacity of the larvae and reduced their ability to neutralize superoxide radicals. Similar reductions in antioxidant enzyme activity have been reported following exposure to nanomaterials; for example, Ref. [75] documented decreased SOD levels in zebrafish exposed to nano-plastics. Modulation of SOD activity is widely reported in zebrafish early life stages following metal exposure, reflecting dynamic regulation of antioxidant defenses that may manifest as either induction or suppression depending on concentration and exposure duration [76,77,78]. Nanoparticles may interfere with cellular redox regulation through interactions with biological membranes, mitochondrial function, or intracellular signaling pathways, leading to altered antioxidant enzyme activity even in the absence of pronounced oxidative damage [79].

Hydrogen peroxide (H_2_O_2_) levels exhibited a significant treatment-specific response (one-way ANOVA: F_2,15_ = 55.729, *p* < 0.0001; Figure 10b). Larvae exposed to Co showed a marked increase in H_2_O_2_ concentrations compared with both the control and MCN groups, suggesting substantial peroxide accumulation and disruption of redox homeostasis. In contrast, MCN alone exposure did not significantly alter H_2_O_2_ levels relative to the control group.

Consistent with peroxide accumulation, lipid peroxidation assessed via MDA content also differed significantly among treatments (one-way ANOVA: F_2,15_ = 7.005, *p* = 0.0071; Figure 10c). Larvae in Co group exhibited significantly higher MDA levels compared with both the control and MCN groups, indicating enhanced oxidative damage to membrane lipids. No significant differences were detected between larvae exposure in control and MCN groups, suggesting that MCN alone did not induce detectable lipid peroxidation under the tested conditions.

The collective exposure to Co resulted in a significant oxidative stress profile, as indicated by a reduction in SOD activity, accompanied by elevated H_2_O_2_ and MDA levels. This coordinated biomarker pattern is indicative of a disruption of redox homeostasis, an accumulation of reactive oxygen species (ROS), and subsequent oxidative damage to membrane lipids. This response is consistent with established mechanisms of metal-induced oxidative stress, whereby transition metals promote ROS generation either directly or indirectly through mitochondrial dysfunction and redox-active pathways. Recent studies on zebrafish have demonstrated that exposure to metals disrupts the redox balance, overwhelms the enzymatic antioxidant capacity, and promotes oxidative injury during early development [76,80,81]. The concurrent decline in SOD activity observed here suggests enzymatic depletion or oxidative inactivation under sustained ROS pressure, thereby limiting superoxide detoxification and favoring downstream peroxide accumulation. Elevated MDA levels serve to further confirm the occurrence of lipid peroxidation as a downstream consequence of excessive ROS formation.

In contrast, MCN-alone exposure led to a reduction in SOD activity without concomitant increases in H_2_O_2_ or MDA levels, suggesting a modulation of antioxidant regulation rather than overt oxidative injury under the tested conditions. This pattern indicates that MCN exposure may have produced a sublethal disturbance in the antioxidant defense system, weakening the capacity of larvae to cope with oxidative challenges without immediately triggering measurable lipid peroxidation. This dissociation between antioxidant enzyme modulation and measurable oxidative damage has been reported for certain chitosan-based nanomaterials, whose biological effects are strongly dependent on formulation, surface chemistry, and dose [29,82]. The absence of significant peroxide accumulation or lipid peroxidation suggests that, at the applied concentration, MCNs do not substantially disrupt redox homeostasis, despite influencing enzymatic antioxidant activity. However, the observed suppression of SOD activity may still represent an important early indicator of oxidative stress susceptibility. A reduced antioxidant capacity may increase the vulnerability of organisms to additional stressors, particularly during sensitive developmental stages [83].

In summary, the integrated biomarker profile identifies cobalt (II) as the primary driver of oxidative imbalance and membrane lipid damage in the present study, underscoring oxidative stress as a central mechanism of metal-induced toxicity during early fish development. These findings underscore the necessity of distinguishing between nanoparticle-induced enzymatic modulation and genuine oxidative injury when evaluating nanomaterial-metal interactions and environmental risk. The assessment of oxidative stress biomarkers was not possible in the Co + MCN group due to the high zebrafish larvae mortality rate at 96 h post-exposure, which precluded the direct mechanistic interpretation of the combined effects. Nevertheless, the reduction in SOD activity observed in the MCN group suggests that MCN exposure may have weakened antioxidant defenses during co-exposure. Such impairment could increase susceptibility to cobalt-induced ROS generation, providing a biologically plausible explanation for inhibition and mortality observed in the Co + MCN treatment. Despite the ambiguity surrounding the precise mechanisms underlying the combined toxicity, the findings suggest a probable contribution of cobalt-driven oxidative disruption to the heightened susceptibility and mortality observed in early life stages.

## 4. Conclusions

This study systematically evaluated the physicochemical performance and biological consequences of magnetite–chitosan nanocomposites (MCN) and cobalt (II) under single- and combined-exposure in zebrafish early life stages. The adsorption data were best described by the Langmuir model, indicating favorable Co^2+^ adsorption by MCN in fish incubation medium (E3), with a maximum experimental adsorption capacity of 20.08 mg g^−1^ and a predicted adsorption capacity (Q_max_) of 29.553 mg g^−1^. Sorption experiments further demonstrated that up to approximately 80% of cobalt (II) could be sequestered at a sorbent-to-metal ratio of 1:50 (*w*/*w*), thereby underscoring the strong binding potential of MCN under controlled batch conditions. However, effective practical application also requires timely separation and recovery of the sorbent after metal binding, as prolonged co-exposure of organisms to metal-loaded nanocomposites may contribute to unintended biological effects. Biological assessment revealed that Co (II) (Co group) reduced hatching success, induced persistent bradycardia, and triggered oxidative stress characterized by decreased SOD activity and elevated H_2_O_2_ and MDA levels. MCN alone did not induce mortality or lipid peroxidation but reduced SOD activity, suggesting modulation of antioxidant regulation without overt oxidative injury. In contrast, combined cobalt and MCN exposure resulted in pronounced delayed mortality, with mortality exceeding 85% at 96 h. The mortality rate observed in this study significantly exceeded that observed under either single exposure, indicating a clear non-additive interaction between cobalt and MCN. Although hatching was partially restored relative to cobalt (II), early neuromotor impairment was observed as reduction in burst duration in locomotor activity. Collectively, these results demonstrate that efficient abiotic sequestration of cobalt (II) does not inherently guarantee biological safety, suggesting a potential dissociation between sorption efficiency and toxicological outcomes. The interactions between nanomaterial and metal can significantly modify toxicity during critical developmental windows of organisms. These findings underscore the limitations of solely relying on sorption efficiency as an indicator of environmental safety. They call for a more comprehensive evaluation framework that incorporates organism-level responses, exposure dynamics, and sorbent recovery strategies into the safe-by-design assessment of remediation nanomaterials. Such integrative assessment is imperative to ensure that nanomaterials designed for environmental remediation do not introduce unintended ecological risks.

## Figures and Tables

**Table 1:** Mössbauer spectral parameters: I—relative area of the hyperfine field distributions for the A and B sublattices and the additional singlet; δ—isomer shift relative to α-Fe; ⟨B〉—average hyperfine field.

Sample	*I*, %	*δ*, mm s^−1^	〈B〉, T	
Magnetite	81	0.32 ± 0.01	45.6	Mag. A (Fe^3+^)
	18	0.67 *	39.5	Mag. B (Fe^2+^ + Fe^3+^)
	1	0.4 *	-	
MCN	87	0.32 ± 0.01	45.9	Mag. A (Fe^3+^)
	12	0.65 *	36.2	Mag. B (Fe^2+^ + Fe^3+^)
	1	0.4 *	-	

*—fixed, I (%) represents the relative spectral area of the fitted Mössbauer components and does not directly correspond to the mass or molar fraction of Fe in the samples.

**Table 2:** Parameters of isothermal models derived from non-linear fit for Co (II) adsorption on MCN in E3 solution.

Isotherm Model	Parameters		*R* ^2^	χ^2^
Freundlich	*1/n*	0.590	0.989	0.603
	*K_F_*	1.225		
Langmuir	*Q_max_*	29.553	0.993	0.392
	*K_L_*	0.017		
	*R_L_*	0.333		
Temkin	*K_T_*	0.723	0.883	6.497
	*B*	3.821		

## Data Availability

The raw data supporting the conclusions of this article will be made available by the authors on request.

## Supplementary Materials

The following supporting information can be downloaded at: https://www.mdpi.com/article/10.3390/biology15080624/s1, Supplementary Figure S1. UV-Vis spectrograms of Co (II) in E3 medium at concentrations of 2 mM (pH = 7.31) and 0.25 mM (pH = 7.15), recorded before and after sorption. Supplementary Figure S2. Sorption of Co (II) from E3 solution using the MCN-30 and desorption in HNO_3_ solutions. Adsorption conditions: 10.375 mg/L Co (II) in E3, pH = 7.2, 1 g/L MCN-30. Contact time 60 min. Desorption: A conditions: 20 mM HNO_3_, pH approximately 1.75, contact time 20-25 min; B conditions: 80 mM HNO_3_, pH approximately 1.15, contact time 20-25 min. The Milli-Q water Type I was used between each procedure to wash the MCN-30 from solutions used. Refs [84,85,86] are cited in the Supplementary Materials.

## Abbreviations

The following abbreviations are used in this manuscript:

MCN	Magnetite–chitosan nanocomposites
SEM	Scanning electron microscopy
TEM	Transmission electron microscopy
ATR-FTIR	Attenuated Total Reflectance-Fourier Transform Infrared spectroscopy
XRD	X-ray Diffraction
