# Supplementary material for: Biological Consequences of Single and Combined Exposure to Magnetite–Chitosan Nanocomposite with Adsorbed Cobalt (II) in Danio rerio
Source: Biology (Basel). 2026 Apr 16;15(8):624. doi: 10.3390/biology15080624 (PMC13113864; doi:10.3390/biology15080624)
Supplement: Supplementary file 1 [file biology-15-00624-s001.zip › biology-4202334-supplementary.pdf]

Supplementary File

Biological consequences of single and combined exposure to magnetite-chitosan nanocomposite with adsorbed cobalt (II) in *Danio rerio*

Sergej Šemčuk[1,2*], Danguolė Montvydienė[2], Renata Butrimienė[2], Aida Bradauskaitė[2], Galina Lujanienė[1], Martynas Talaikis[1], Kęstutis Mažeika[1], Vidas Pakštas[1], Justas Lazutka[3], Živilė Jurgelėnė[2*]

1 State Research Institute Center for Physical Sciences and Technology (FTMC), Savanorių ave. 231, LT-02300 Vilnius, Lithuania
2 State Scientific Research Institute Nature Research Centre (NRC), Akademijos st. 2, LT-08412 Vilnius, Lithuania
3 Vilnius University, Life Sciences Centre, Institute of Biotechnology, Saulėtekio al. 7, 0222, Vilnius, Lithuania.
* Correspondence: sergej.semcuk@ftmc.lt and zivile.jurgelene@gamtc.lt

The spectrograms were recorded using a UV/Vis spectrophotometer (Analytik Jena Specord 200 Plus, Burladingen, Germany) in range from 190 nm to 1100 nm to analyze the speciation of the dissolved cobalt.

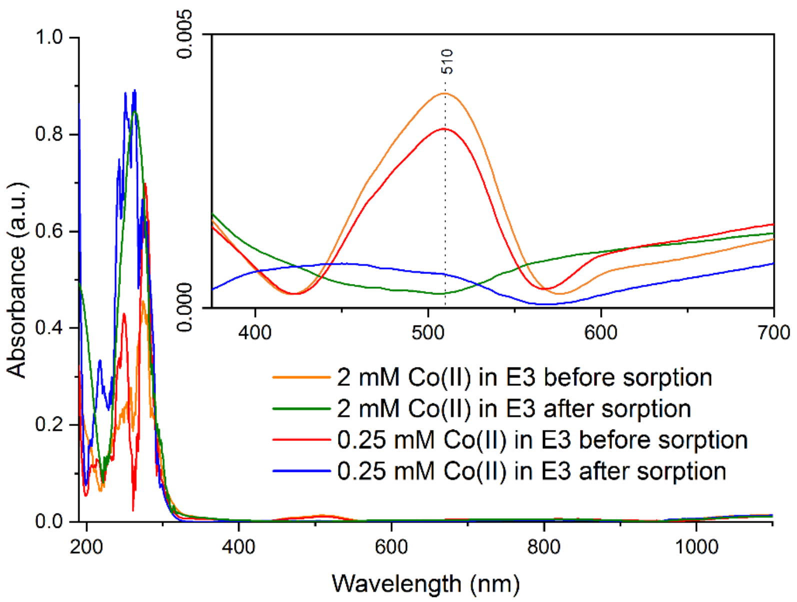

Supplementary Figure S1. UV-Vis spectrograms of Co (II) in E3 medium at concentrations of 2 mM (pH=7.31) and 0.25 mM (pH=7.15), recorded before and after sorption.

The recorded UV-Vis spectra are presented in Figure SF1. The results show an absence of the characteristic Co (III) absorption peaks at 340 and 460-470 nm [84], as well as no peaks in the 600-700 nm range characteristic for the tetrahedral $[CoCl4]^{2-}$[85]. Furthermore, the absence of $Co(OH)_2$ precipitation in all used solutions confirms that the $CoCl_2 \cdot 6H_2O$ salt was fully dissolved. In the solution, cobalt remains in its divalent state as Co (II) and $[Co(H_2O)_6]^{2+}$, that is evidenced by the small peaks at 510 nm [86]. These signals are of low intensity due to the relatively low cobalt concentrations, and their absence after adsorption is likely attributable to the detection limits of the spectrophotometer.

The adsorption and desorption experimental data were detected by measuring the concentration changes of Co (II) in the used solution. The solutions were analyzed by optical emission spectrometry using inductively coupled plasma (ISP-OES, Optima7000DV, Perkin Elmer, USA). Standardized solutions of 0.100 mg/L; 1.00 mg/L and 10.00 mg/L (standard solution N8 Perkin Elmer, USA) were used for instrument calibration. Wavelength Co 228.616 nm.

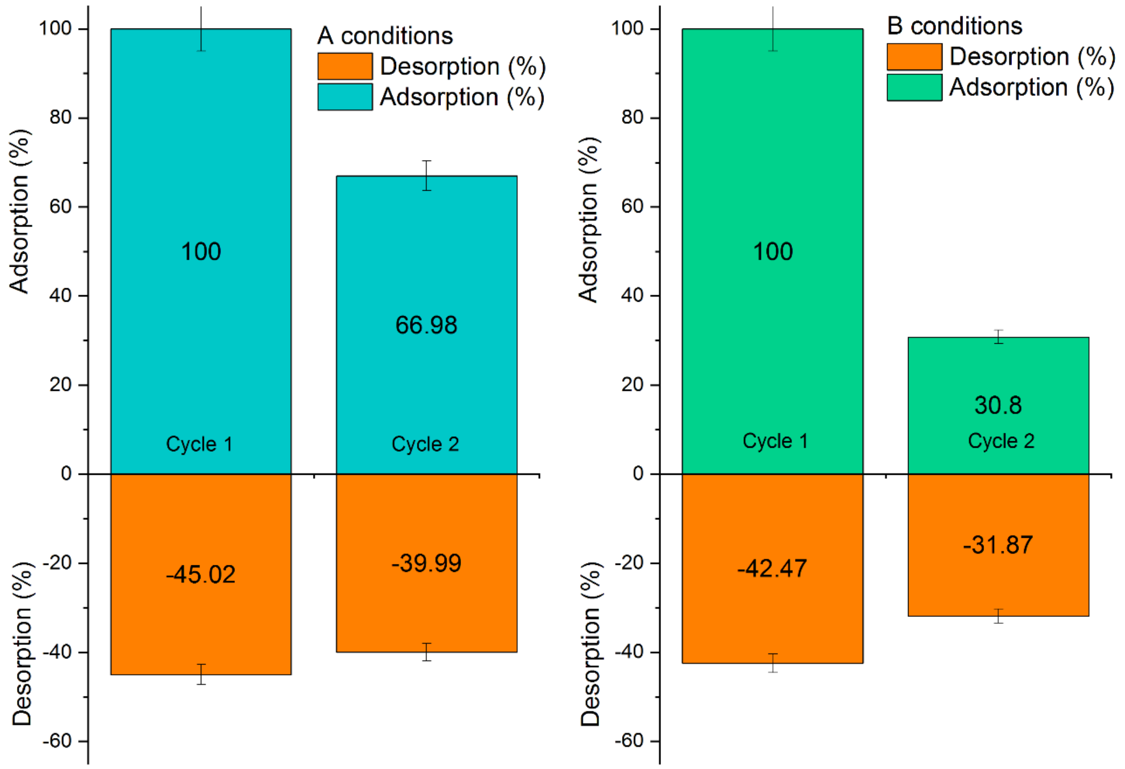

Supplementary Figure S2. Sorption of Co (II) from E3 solution using the MCN-30 and desorption in HNO_3 solutions. Adsorption conditions: 10.375 mg/L Co (II) in E3, pH = 7.2, 1 g/L MCN-30. Contact time 60 min. Desorption: A conditions: 20 mM HNO_3, pH approximately 1.75, contact time 20-25 min; B conditions: 80 mM HNO_3, pH approximately 1.15, contact time 20-25 min. The Milli-Q water Type I was used between each procedure to wash the MCN-30 from solutions used.
